# Rapid and Sensitive Quantification of Osimertinib in Human Plasma Using a Fully Validated MALDI–IM–MS/MS Assay

**DOI:** 10.3390/cancers12071897

**Published:** 2020-07-14

**Authors:** Margaux Fresnais, André Roth, Kathrin I. Foerster, Dirk Jäger, Stefan M. Pfister, Walter E. Haefeli, Jürgen Burhenne, Rémi Longuespée

**Affiliations:** 1 Department of Clinical Pharmacology and Pharmacoepidemiology, Heidelberg University Hospital, Im Neuenheimer Feld 410, 69120 Heidelberg, Germany; Margaux.Fresnais@med.uni-heidelberg.de (M.F.); AR_Roth@web.de (A.R.); Kathrin.Foerster@med.uni-heidelberg.de (K.I.F.); Walter-Emil.Haefeli@med.uni-heidelberg.de (W.E.H.); Juergen.Burhenne@med.uni-heidelberg.de (J.B.); 2 German Cancer Consortium (DKTK)-German Cancer Research Center (DKFZ), Im Neuenheimer Feld 280, 69120 Heidelberg, Germany; 3 Department of Medical Oncology, National Center for Tumor Diseases Heidelberg, Heidelberg University Hospital, Im Neuenheimer Feld 460, 69120 Heidelberg, Germany; dirk.jaeger@med.uni-heidelberg.de; 4 Hopp Children’s Cancer Center, NCT Heidelberg (KiTZ), Im Neuenheimer Feld 460, 69120 Heidelberg, Germany; s.pfister@kitz-heidelberg.de; 5 Division of Pediatric Neurooncology, German Cancer Research Center (DKFZ), German Cancer Consortium (DKTK), Im Neuenheimer Feld 280, 69120 Heidelberg, Germany

**Keywords:** tyrosine kinase inhibitors, osimertinib, quantification, mass spectrometry, MALDI

## Abstract

The third-generation tyrosine kinase inhibitor (TKI), osimertinib, has revolutionized the treatment of patients with non-small cell lung carcinoma with epidermal growth factor receptor (EGFR)-activating mutation, and resistant to first- and second-generation TKIs. Osimertinib is now also proposed as a first-line therapy, thus extending the scope of applications in lung oncology. Personalized medicine approaches are still necessary to monitor if patients are exposed to adequate concentrations of osimertinib during their treatment. It would also help to understand the appearance of new resistances in patients after several months of dosing with osimertinib. Liquid chromatography–tandem mass spectrometry (LC–MS/MS) is currently the gold standard for the quantification of drugs in plasma enabling pharmacokinetic analyses and patient monitoring. In the present study, we propose an alternative to LC–MS/MS methods for the rapid and sensitive quantification of osimertinib in plasma using matrix-assisted laser desorption/ionization (MALDI) –MS. The presented assay requires only 3 min per sample for their preparation, analysis, and data extraction, and less than 3 h for quantification. A lower limit of quantification (LLOQ) of 5 ng/mL in plasma was retrieved. The method was fully validated, following the guidelines of the US Food and Drug Administration (FDA) and the European Medicines Agency (EMA) for bioanalytical method validation. The present developments prove the importance to consider alternative MS assays for time-efficient quantification of small molecule inhibitors in plasma in the context of personalized medicine for targeted therapies.

## 1. Introduction

Small molecular inhibitors and, in particular tyrosine kinase inhibitors (TKIs), are gaining increasing importance among oncological therapies. In non-small cell lung cancer (NSCLC), progresses are being made to circumvent the mechanism of resistance to other TKIs such as gefitinib, erlotinib, afatinib, and dacotinib, due to secondary mutations of epidermal growth factor receptor (EGFR) kinase domain. In 2017, the third-generation TKI, osimertinib, was approved by the US Food and Drug Administration (FDA) for the treatment of NSCLC patients with the EGFR-activating mutation T790M. Improved median progression-free survival, median duration of response, and survival rates at 18 months of osimertinib over standard EGFR-TKIs for EGFR mutation-positive advanced NSCLC were recently reported in the FLAURA study [1]. Osimertinib is currently recommended as first-line therapy for metastatic NSCLC with EGFR mutation [2]. Despite the great performance of osimertinib, lower efficacy is observed with patients with poor Eastern Cooperative Oncology Group performance status [3]. Acquired resistance to osimertinib also occurs and investigations are necessary to understand the underlying mechanisms to these resistances [4]. Finally, adverse effects such as rash and diarrhea can be observed, which are more frequent with exposure to higher concentrations of osimertinib [5]. Methods for the quantification of osimertinib in plasma are necessary to monitor that patients are exposed to a concentration range that is sufficient for pharmacological effect while resulting in reduced adverse effects. Investigating the mechanism of resistance would also imply monitoring if resistant patients are still exposed to the drug.

Mass spectrometry (MS) is adequate for the absolute quantification of drugs in plasma and among all MS platforms, liquid chromatography–tandem MS (LC–MS/MS) is currently the gold-standard. However, sample preparation and analyses can be relatively time-consuming using LC–MS/MS compared to desorption methods such as matrix-assisted laser desorption/ionization (MALDI) [6,7] and desorption electrospray ionization (DESI) [8]. These latter MS sources usually require a minimum of sample preparation because ions are formed directly from solid samples on surfaces. Molecular complexity within these solid preparations usually does not cause problems of instrumental contamination as with LC systems, where strong carry-over effects can be observed between runs when too complex and too concentrated samples are loaded. Furthermore, only dedicated solvents such as methanol and acetonitrile are compatible with LC columns for separations, while samples dried from any solvent can directly be analyzed by MALDI or DESI. However, restricted sample preparation often takes place at the expense of selectivity when instruments with relatively low resolving power are used. We recently illustrated the merits of MALDI–ion mobility (IM)–MS profiling for the quantification of drugs in tissue sections [7]. Direct quantification by MALDI indeed confers important advantages of rapidity and low input sample consumption. Additionally, the ion mobility spectrometry (IMS) mode conferred a considerable asset of selectivity for the post-acquisition signal filtering and subsequent quantification of drugs separated in the gas phase [7]. IM–MS-based data acquisition also mimics LC–MS-based techniques. Indeed, areas of signals from compounds separated in gas phase can be measured from mobilograms with IMS, just like areas of signals from compounds eluted in liquid chromatography can be measured from chromatograms with LC–MS. In this latter study, we illustrated the possibility to validate MALDI–MS methods for on-tissue quantification of small compound xenobiotics, according to the FDA and European Medicines Agency (EMA) guidelines for bioanalytical method validation [9,10]. Although MALDI appears as the most adequate ion source for the analysis of solid samples, it also confers the advantage of rapidity for the analyses of liquid samples deposited on surfaces, such as preparations from plasma. However, only few studies exploited MALDI for absolute quantification in plasma. Bile acids [11], 25-hydroxyvitamin D3 [12], human immunodeficiency virus (HIV) protease inhibitors [13], CIGB-300 antitumor peptide [14], and methotrexate [15] were already successfully quantified in human plasma, and the two latter studies were either partially or fully validated according to the FDA guidelines for bioanalytical validation.

In the present work, we elaborated a MALDI–IM–MS/MS method for the rapid and sensitive quantification of osimertinib in human plasma. We exploited the different features of an intermediate pressure orthogonal acceleration (oa)-quadrupole (Q)–IMS–time-of-flight (TOF) mass spectrometer and combined it with an adapted sample preparation procedure to allow for the quantification of osimertinib in plasma down to 5 ng/mL in less than 3 h, including sample preparation, analyses, data extraction, and creation of the calibration curve. The developed method was fully validated according to the guidelines of the FDA and EMA for bioanalytical method validation. The validated method was then applied to samples from a clinical study, and our bioanalytical assay was cross-validated using LC–MS/MS results obtained on this study.

## 2. Results

The goal of the study was to establish a rapid method for the quantification of osimertinib in plasma from patients treated for NSCLC with EGFR mutation using MALDI–IM–MS/MS and to validate it according to the guidelines of the FDA and EMA for bioanalytical method validation. We aimed to provide an alternative to LC–MS/MS for faster sample preparation and analysis turnaround that is sensitive enough to measure therapeutic concentrations in plasma. We recently established a rapid assay for the quantification of drugs in tissue sections using MALDI–IM–MS/MS profiling and extraction of IMS-generated data, where ion mobility was used as a pseudo-chromatographic method for the separation of analyte ions from endogenous interfering ions [7]. We aimed to verify that the developed assay was adaptable to plasma samples and would be suitable for the quantification of osimertinib. As described in the Materials and Methods Section 4.6 MS Parameters, we used a method designed for the quantification of fragments specific to the targeted xenobiotic in the biological matrix. First, the method allows us to perform a first signal filtering of the full ion content of plasma samples by selecting only parent ions in a limited mass range (here around mass/charge (*m*/*z*) 500) before fragmentation and subsequent IM separation. Second, targeting fragments allows us to improve sensitivity and selectivity by focusing the measurements on a signature of the compound of interest. In the chosen method, fragments are then used as specific molecular signatures differentiating the drug from its internal standard (IS) and fragments of endogenous interfering ions. Consequently, the first step of the development consisted in the characterization of MS/MS fragments of osimertinib and the IS, [^13^C,^2^H_3_]-osimertinib. Second, sample preparation procedures were tested to allow for the homogenous crystallization of the samples and thus to promote the obtention of a homogenous signal during MS acquisition. Third, calibration curves were created and validation batches were performed following the regulatory guidelines. The assay was finally applied to the quantification of osimertinib in plasma from dosed patients with NSCLC and cross-validation of the method was performed using LC–MS/MS data from the same patients.

### 2.1. Method Development

As described in the Materials and Methods Section, we used a method designed for the quantification of fragments of the targeted xenobiotic, osimertinib, in the biological matrix. The different steps of assay development were as follows:

1. Characterizing MS/MS fragments of osimertinib and of the IS, [^13^C,^2^H_3_]-osimertinib using MALDI–MS/MS.
2. Testing sample preparation procedures yielding homogenous crystallization of the samples with the MALDI matrix.
3. Evaluating the osimertinib signal in plasma for further quantification.
4. Creating calibration curves of osimertinib normalized by the IS, [^13^C,^2^H_3_]-osimertinib.

#### 2.1.1. Characterization of MS/MS Fragments of Osimertinib and [^13^C,^2^H_3_]-Osimertinib

**Figure 1:**
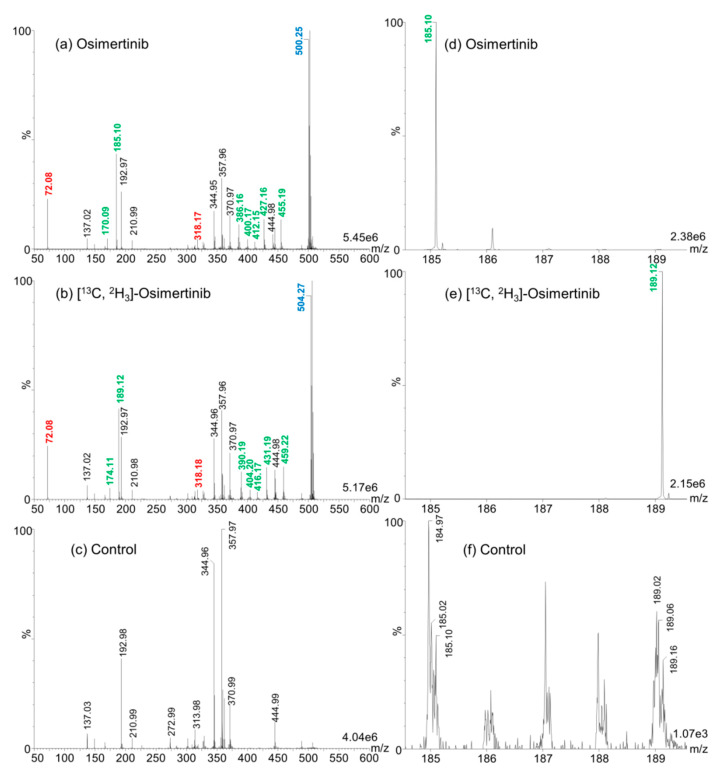
Differential matrix-assisted laser desorption/ionization-ion mobility-tandem mass spectrometry (MALDI-IM-MS/MS) fragments between osimertinib and [^13^C,^2^H_3_]-osimertinib. (**a**) MALDI-IM-MS/MS spectrum of osimertinib. (**b**) MALDI-IM-MS/MS spectrum of [^13^C, ^2^H_3_]-osimertinib. (**c**) MALDI-IM-MS/MS spectrum of 2,5-dihydroxybenzoic acid (2,5-DHB), used as control. The mass/charge (*m*/*z*) values in blue correspond to the native compounds. Red *m*/*z* values correspond to fragments that were found in common between osimertinib and [^13^C,^2^H_3_]-osimertinib. Green *m*/*z* values correspond to differential fragments. Black *m*/*z* values represent peak fragments found in osimertinib, [^13^C,^2^H_3_]-osimertinib, and control solutions. (**d**–**f**) MALDI-IM-MS/MS spectra in the *m*/*z* range 184 to 190 of osimertinib, [^13^C,^2^H_3_]-osimertinib, and control, respectively. The maximum intensities are given in arbitrary units at the bottom right of each spectrum.

For the method development, 2,5-dihydroxybenzoic acid (2,5-DHB) was used as MALDI matrix given its universality for the ionization of small compound drugs. We applied the MALDI–IM–MS/MS method described in the Materials and Methods Section for the determination of differential fragments between osimertinib and [^13^C,^2^H_3_]-osimertinib. We could discriminate seven differential fragments including a major one at mass/charge (*m*/*z*) 185 for osimertinib and *m*/*z* 189 for [^13^C,^2^H_3_]-osimertinib, as shown in Figure 1. These fragments seem uniquely observed in MALDI and were not previously described using electrospray ionization (ESI) [16,17,18,19]. We used these fragments for quantification in the further development steps.

#### 2.1.2. Sample Preparation

The major advantage of MALDI–MS methods compared to LC–MS is the possibility to reduce time for sample preparation. However, the lack of chromatographic separation can greatly hamper the specificity of the measurements and consequently, sensitivity. For developments for MALDI–MS-based drug quantification, it is mandatory to adapt as much as possible sample preparation procedures to reduce the impact of interfering endogenous compounds of the investigated biological matrix. Therefore, a two-step liquid–liquid extraction (LLE)-based procedure used for LC–MS applications [20] was adapted for MALDI applications. A protocol using tert-butyl methyl ether (TBME) was optimized for the simultaneous extraction and concentration of osimertinib from plasma, as described in the Materials and Methods Section 4.5 Calibration Standard and Quality Control Sample Preparation. Compared to common LLE methods, we aimed at reducing the volume of TBME for LLE from plasma samples in order to retrieve the drug and the IS concentrated in a low volume of the upper layer (organic phase). The drug and IS concentrated in a small volume can then directly be deposited on the MALDI metal targets for further analyses.

**Figure 2:**
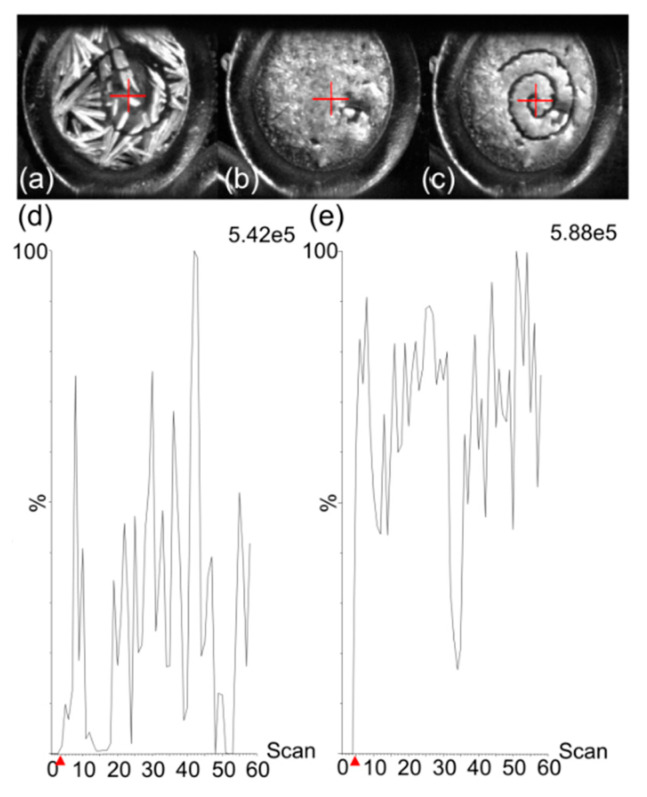
(**a**) Morphology of a sample with 2,5-dihydroxybenzoic acid (2,5-DHB) deposited after the deposition of a liquid–liquid extraction (LLE) extract, after analysis. Morphology of a sample after the deposition of a LLE extract on metal target pre-spotted with 2,5-DHB (**b**) before and (**c**) after analysis. (**d**) Total ion current (TIC) from sample (**a**). (**e**) TIC from sample (**c**). The maximum intensities are given in arbitrary units at the top right of each TIC. Red arrows on the x-axis correspond to the time of laser start.

An additional drawback of MALDI–MS for quantification purposes is the heterogenous distribution of crystals of a large panel of matrices over analysis surfaces after drying of 1 µL droplets. Contrarily to LC–ESI–MS approaches where the signal is very stable during the analysis, crystal heterogeneity using numerous MALDI matrices can cause important signal variations during the analysis. Because molecules are present in—and are desorbed from—MALDI crystals, crystallization heterogeneity can reduce signals when automatic methods and defined geometric patterns are used for laser motion. This is the case for 2,5-DHB that crystallizes at the outer edges of deposition spots (Figure 2a). We tested two methods to leverage this drawback (see Section 4.5 Calibration Standard and Quality Control Sample Preparation). These two methods differed in depositing 2,5-DHB before or after the deposition of the LLE extract. As displayed in Figure 2, the use of a spiral-shaped geometrical pattern for analysis only allowed us to reach a limited proportion of matrix crystals when 2,5-DHB was deposited after deposition of the LLE-extracted sample (Figure 2a). This resulted in low and unstable total ion current (TIC) over the 60 s of analysis (Figure 2d). The deposition of the LLE extract containing TBME, the drug, and the IS on crystals of pre-spotted 2,5-DHB allowed us to resolubilize the 2,5-DHB crystals and to obtain a quasi-fully homogenous deposition after recrystallization onto the spot of the metal target (Figure 2b). The spiral-shaped geometrical pattern used for further analysis (Figure 2c) allowed us to obtain an overall higher and more stable TIC (Figure 2e). Because the sample preparation approach provided deposits that were adequate with downstream MS acquisition methods, we used this method for the detection and quantification of osimertinib in plasma.

#### 2.1.3. Evaluation of the Signal of Osimertinib in Plasma

**Figure 3:**
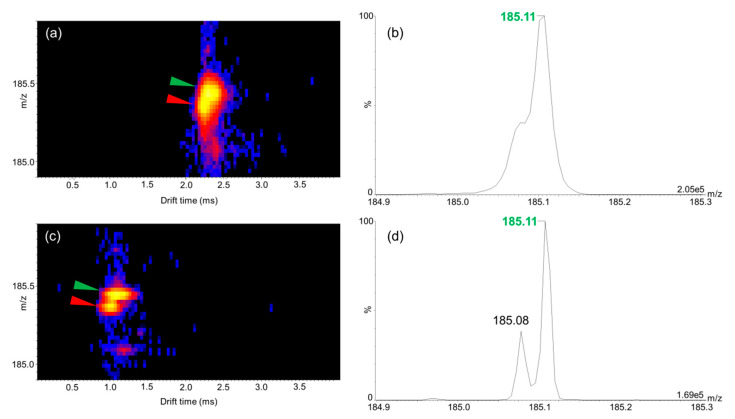
Matrix-assisted laser desorption/ionization–ion mobility–tandem mass spectrometry (MALDI–IM–MS/MS) analyses of liquid–liquid extraction extracts from plasma spiked with 1000 ng/mL of osimertinib. (**a**) Mass /charge (*m*/*z*) vs. drift time (DT) map in *m*/*z* 184.9 to 185.9 and DT 0 to 4 ms ranges in sensitivity mode. (**b**) Mass spectrum in *m*/*z* 184.9 to 185.3 range in sensitivity mode. (**c**) *m*/*z* vs. DT map in *m*/*z* 184.9 to 185.9 and DT 0 to 4 ms ranges in resolution mode. (**d**) Mass spectrum in *m*/*z* 184.9 to 185.3 range in resolution mode. In (**a**,**c**), green arrows indicate the fragment ion of osimertinib and red arrows the interfering fragment ion. The maximum intensities are given in arbitrary units at the bottom right of each spectrum in (**b**,**d**).

In order to evaluate whether interfering fragment ions of endogenous molecules from the plasma were present, we evaluated two-dimensional (2D) *m*/*z* vs. drift time (DT) maps. The data informed us that an interfering fragment was visible in the close vicinity of the *m*/*z* 185.1 osimertinib fragment with a ΔDT of 0.04 ms and a Δ*m*/*z* of 0.03 (Figure 3). The mobility separation did not allow us to fully resolve the two mobility peaks of osimertinib and the interfering ion fragments. For the developments described thus far, the instrumentation was used with a single reflection step of the ions in the TOF tube (“V” mode or sensitivity mode). This mode allows for a relatively higher sensitivity but lower mass resolution when compared to the other modes of analyses of the instrument. Another mode of analysis allows for measurements with higher mass resolving power using two reflections through the TOF tube (“W” mode or resolution mode). We hypothesized that using the resolution mode would have a higher impact on the separation of osimertinib from the interfering ion fragment than ion mobility separation itself. This was verified after analysis in resolution mode, as displayed in Figure 3. In this case, the gain in peak resolution helped to reach a better sensitivity by allowing us to better discriminate the osimertinib peak from the interfering signal. Therefore, we used the resolution mode of the instrument for further developments.

#### 2.1.4. Calibration Curve of the Response of Osimertinib Normalized by [^13^C,^2^H_3_]-Osimertinib

Calibration curves were performed over more than three orders of magnitudes from 5 to 1000 ng/mL in pre-validation batches including four quality control (QC) levels: lower limit of quantification (LLOQ), low QC (LQC), middle QC (MQC), and high QC (HQC) (Table 1). As previously described [7], we used two approaches for data extraction, using *m*/*z* intensities in combined mass spectra from TIC (MassI) or using automatically integrated areas of extracted mobility peaks of the compounds of interest (MobA). Calibration curves computed using 1/x^2^ weighing displayed determination coefficient r^2^ around 0.98 using MassI and >0.985 using MobA methods. The MassI method showed slightly lower linearity performances and greater bias in accuracy measurements for calibration standard (CAL) and QC samples. Furthermore, the MobA data extraction method is more similar to the data extraction method in LC–MS/MS in multiple reaction monitoring (MRM) mode where the LC peaks of the specific transitions are integrated to calibrate and quantify the samples. The MobA method was then used for the method validation.

### 2.2. Method Validation

The MALDI–IM–MS/MS assay for the quantification of osimertinib with MobA data extraction was validated following the FDA and EMA guidelines for bioanalytical method validation [9,10]. It is also important to note that, in parallel to MobA, the MassI data extraction method was also tested for the method validation, but using MassI, it was not possible to successfully validate the method following the regulatory guidelines.

**Figure 4:**
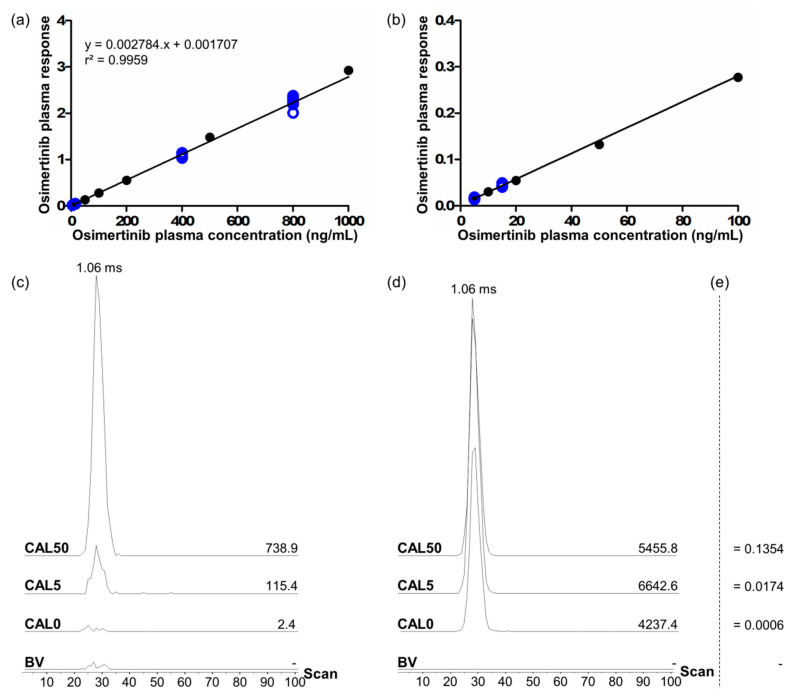
Calibration curve obtained for one of the validation batches (**a**) from 5 to 1000 ng/mL and (**b**) on a zoomed range from 5 to 100 ng/mL showing the mean responses (arbitrary units) obtained for the different calibration levels (full black circles, standard deviations are displayed but too low to be visible on the graphs) and the responses obtained for each individual quality control (QC) replicate (empty blue circles). Extracted ion mobilograms of (**c**) osimertinib (mass/charge (*m*/*z*) range: 185.094 to 185.121) and (**d**) [^13^C,^2^H_3_]-osimertinib (*m*/*z* range: 189.125 to 189.140) for a blind value sample (BV), a CAL0 sample (BV with internal standard), a CAL5 sample (LLOQ level), and a CAL50 sample (50 ng/mL osimertinib). On the right of each baseline the peak area in arbitrary units is given; the computed responses corresponding to each sample are given in (**e**).

Validation included four batches on four different days. A typical calibration curve with associated QC samples, and obtained extracted ion mobilograms and responses for blind value (BV, control plasma sample), CAL0 (BV spiked with IS), LLOQ (CAL5), and CAL50 samples (Table 1) are shown in Figure 4. Full validation results are summarized in Table 2.

The LLOQ was set to 5 ng/mL and sensitivity was shown across the first three validation batches using eight technical replicates of LLOQ-level QC samples. As recommended in the guidelines for LLOQ level, within-batch and batch-to-batch precisions were <20% coefficient of variation (CV), with within-batch precisions between 9.8 and 12.6% CV, and a batch-to-batch precision of 11.9% CV. Similarly, within-batch and batch-to-batch accuracies fell into the recommended ±20% bias limits, with within-batch accuracies between −3.4 and 4.6% bias, and a batch-to-batch accuracy of 0.7% bias. Over the full validation procedure, four calibration curves were computed using MassLynx software by automatic integration of extracted mobility peaks (ApexTrack processing algorithm). All four curves were drawn between 5 and 1000 ng/mL using a linear regression model with 1/x^2^ weighting. Linearity was proven for every batch on at least seven non-zero calibration levels with r^2^ determination coefficients ≥0.988, and >75% of calibration standards within the ±15% bias accuracy limits and below the 15% CV limit between replicates. All validation runs passed acceptance criteria (15% CV for precision and ±15% bias for accuracy) with within-batch accuracies between −5.9 and 3.5% bias, within-batch precisions between 1.8 and 9.1% CV, batch-to-batch accuracies between 3.5 and 8.4% bias, and batch-to-batch precision between −1.7 and 2.2% CV on ≥six replicates per level for the three QC levels (LQC, MQC, and HQC).

The specificity of the method was also proven for osimertinib and [^13^C,^2^H_3_]-osimertinib, using blank plasma samples (BV samples) from six different individuals. In these samples, no targeted signal was detected for osimertinib and the detected signal for [^13^C,^2^H_3_]-osimertinib was <0.4% of the mean IS signal from calibration standards and QC samples (Figure 4c,d).

Over the three QC levels (LQC, MQC, and HQC), extraction yielded a mean recovery of 53.4% with an overall precision of 13.4% CV. Matrix effects were tested on all three levels and shown to be negligible with an IS-normalized matrix factor (MF) of 102.5%, 98.1%, and 99.4% at the LQC, MQC, and HQC levels, respectively, with precisions between the four replicates between 7.1 and 9.9% CV (mean IS-normalized MF of 100.0% with a precision of 8.3% CV).

Furthermore, when analyzing 2,5-DHB spots deposited with a blank upper layer mix (see Section 4.8.3) on previously cleaned CAL500 and CAL1000 positions on the MALDI target, no significant or reproducible carry-over occurred with mean targeted signals always <17% of the lowest osimertinib signal at the LLOQ level (below the 20% limit specified in the regulatory guidelines) and <0.4% of the mean IS signal from calibration standards and QC samples (below the 5% limit specified in the regulatory guidelines).

### 2.3. Cross-Validation of the MALDI–IM–MS/MS Assay

To cross-validate the MALDI–IM–MS/MS assay following FDA and EMA regulation guidelines, it was applied to the quantification of osimertinib in clinical samples from the PROMISE clinical study (see Section 4.3 Clinical Samples). Twenty-five clinical samples from three patients were analyzed and compared to LC–MS/MS data previously obtained with a fully validated assay (see Section 4.9 LC–MS/MS-Based Quantification of Osimertinib).

**Figure 5:**
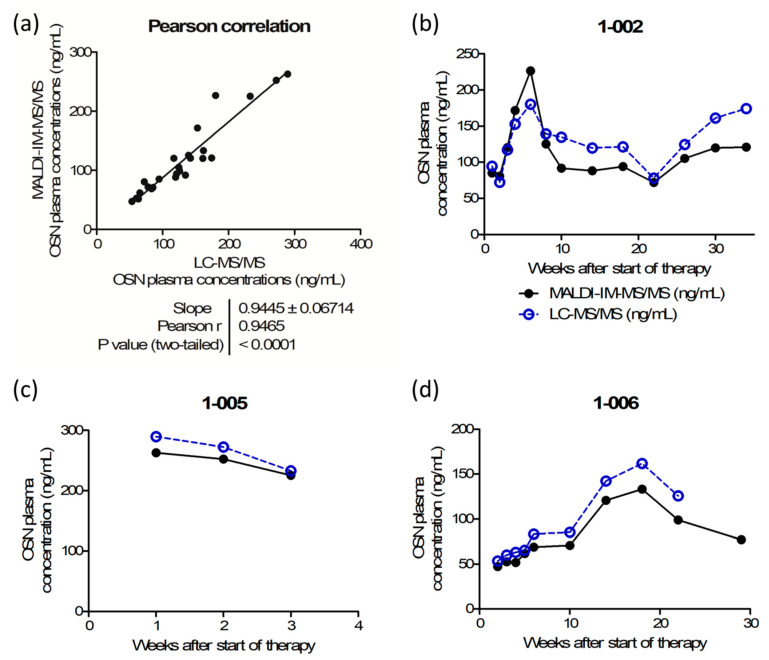
Cross-validation of the matrix-assisted laser desorption/ionization–ion mobility–tandem mass spectrometry (MALDI–IM–MS/MS) assay. (**a**) Pearson correlation of quantification results from clinical samples showing a significant correlation between the results obtained with the MALDI–IM–MS/MS and the liquid chromatography (LC)–MS/MS analytical methods (*r* = 0.9465, *p* value < 0.0001). Evolution of osimertinib plasma concentration over the first weeks of therapy for three patients: (**b**) 1-002, (**c**) 1-005, and (**d**) 1-006 with MALDI–IM–MS/MS results displayed as full black circles and LC–MS/MS results displayed as empty blue circles.

Cross-validation using 25 incurred samples was validated with 72% of the compared samples being conform to the regulatory guidelines, i.e., the difference between the mean concentrations obtained with LC–MS/MS and with MALDI–IM–MS/MS should be within the limit of ±20% of the mean. A Pearson product–moment correlation test confirmed the significance of the correlation between the results obtained using MALDI–IM–MS/MS and LC–MS/MS assays with a Pearson correlation coefficient of *r* = 0.9465 and a *p*-value < 0.0001, as shown in Figure 5a. In addition, the very low variations observed between technical duplicates of a given sample (<11% CV) highlight the repeatability of the MALDI method.

Figure 5b–d depict osimertinib plasma concentrations over time of three patients with NSCLC, showing good alignment of MALDI–IM–MS/MS and LC–MS/MS values in all individuals.

## 3. Discussion

MALDI–MS presents the principal advantage compared to LC–MS-based assays to allow for more flexibility regarding sample preparation because of the absence of chromatographic separation. Indeed, complex samples can be analyzed from any solvent directly after dryness, without hampering instrumental integrity. This advantage has been poorly exploited in the field of clinical pharmacology for drug quantification because of the crystal properties of the MALDI matrices. The most universal MALDI matrix for the ionization of small compounds is 2,5-DHB, which forms long crystals at the edge of droplets deposited on surfaces. As we illustrated in our work, after crystallization, 2,5-DHB deposits leave large crystal-free areas where ionization is not possible. This feature is a considerable drawback for method automatization because common geometrical patterns for sample motion do not allow the targeting of crystals for the analysis. Large variations in signal intensities during a single analysis result in lower summed *m*/*z* intensities of the compound of interest and consequently result in higher LOQs.

In this project, we aimed to adapt a MALDI–MS-based assay for the rapid and sensitive quantification of osimertinib in plasma. MALDI ionization conferred the advantage to obtain after fragmentation a larger major fragment at *m*/*z* 185.1 than with the presented ESI-MS method where a major fragment at *m*/*z* 72.1 is observed.

Our first goal in this study was to solve the problem of heterogeneous crystallization and take advantage of the flexibility conferred by MALDI–MS for sample preparation in a single experiment. We established a rapid two-step protocol of LLE to concentrate the drug in a small volume of TBME. LLE-based procedures for LC–MS/MS using TBME necessitate sample dryness before resuspension in adequate buffers for downstream LC–MS/MS analyses. In the present protocol, the extracted upper layer solution, principally composed of TBME containing osimertinib, [^13^C,^2^H_3_]-osimertinib, and endogenous compounds from plasma, was directly deposited on 2,5-DHB crystals. This TBME-based solution appeared ideal for 2,5-DHB crystals dissolution and recrystallization in a more homogeneous layer containing the compounds to quantify. Overall, approximately 15 s per sample are necessary for preparation and only 1 min of analysis per sample was necessary to obtain the MALDI–IM–MS/MS data using a geometric sample motion. Altogether, the full process, including sample preparation, MS analysis, and data extraction was performed in approximately 3 min per sample. The procedure applied to the creation of calibration curves of osimertinib over more than three orders of magnitude, together with QC sample analysis, is performed in 2 h and allows us to reach an LLOQ down to 5 ng/mL.

Once this protocol was established, we aimed to validate it through a series of validation batches following the guidelines of the FDA and EMA for bioanalytical method validation. Particular considerations were taken for the calculation of recovery and matrix effect, integrating the nature of the extraction solutions that are directly used for MALDI–MS analyses, the dilution effect when spiking compound solutions after extraction, and the ionization variations between analytical replicates inherent to the MALDI process. In the present workflow, the normalization by the IS corrects technical variations during sample preparation but also large ionization variations induced by the MALDI process. Using these particular considerations, we could fully validate the MALDI–IM–MS/MS assay for osimertinib quantification in plasma. This demonstration of method validation represents an important advance in the field of pharmacological analytics to introduce MALDI–MS within the panel of methods for the quantification of small compound inhibitors in biological matrices. As we introduced it formerly [7], the use of IM–MS allowed for a LC–MS-like data integration for drug quantification purposes. Interestingly, methods using ion mobility peak areas (MobA) for data extraction allowed for the validation of analytical batches, contrarily to methods using peak intensities (MassI). In addition to our former study applied to tissue sections [7], the present study stresses the important role that ion mobility separation may play for drug quantification when desorption methods are used.

In parallel, we also cross-validated the MALDI–IM–MS/MS assay using LC–MS/MS data obtained on the same analyzed clinical samples. The significant correlation shown between the results of both methods confirmed the potential of the workflow within the panel of MS methodologies for the quantification of small compound inhibitors in plasma. These data also prove that this rapid and sensitive MALDI–IM–MS/MS method can readily be used to monitor the circulating concentration of osimertinib in plasma from patients over weeks of therapy with the same reliability as LC–MS/MS methods.

In the future, add-in developments, improvements, and adaptations of the method can be envisioned. For instance, multiplexing methods could be created for the simultaneous quantification of different drugs from the very same sample when drug combinations are used for NSCLC patients. Because only a portion of the deposits are consumed after the 60 s of analysis, additional MS measurements could be performed when multiplexing methods would not be applicable. Given the relatively large volume of extraction solution available from further deposition, it could also be envisioned to scale down the volumes of sample and solutions for sample preparation for MALDI–MS-based quantification of drugs in situations where the available quantity of biological matrix is critical, such as in pediatric therapies [21].

Altogether, these developments proved that the drawbacks inherent to MALDI–MS for the quantification of small compound inhibitors in liquid biological matrices can be overcome with adapted rapid sample preparation procedures. Once adequate workflows are established, MALDI–MS and, in particular MALDI–IM–MS, represent a complementary and competitive approach within the panels of analytical options for drug quantification in biological matrices.

## 4. Materials and Methods

### 4.1. Chemical Reagents

MS-grade water, solvents, and formic acid (FA) were purchased from Biosolve Chimie SARL (Dieuze, France). Red phosphorus and 2,5-DHB, trifluoroacetic acid (TFA) were purchased from Sigma-Aldrich (Darmstadt, Germany). Osimertinib and [^13^C,^2^H_3_]-osimertinib were purchased from Alsachim (Illkirch, France).

### 4.2. Plasma Sample Collection

Blank plasma samples from healthy individuals were obtained from the local blood bank (IKTZ Heidelberg GmbH, Heidelberg, Germany) for research purposes.

### 4.3. Clinical Samples

Study samples were obtained within the PROMISE study regrouping patients treated for NSCLC with EGFR mutation. The study was performed following the principles of good clinical practice (GCP) and in accordance with the ethical principles described in the applicable version of the Declaration of Helsinki. The study was approved by the responsible Ethics Committee of the Medical Faculty of Heidelberg University (Ethical vote S-710/2017). All patients were fully informed about the trial and gave their written consent prior to any study procedures. Samples from three patients maintained on 80 mg osimertinib orally once daily, were analyzed within this work: 1-002 (13 samples from week 1 after the start of therapy to week 34), 1-005 (three samples for weeks 1, 2, and 3 after start of therapy), and 1-006 (ten samples from week 1 after start of therapy to week 29, among which only the first nine samples were also analyzed by LC–MS/MS). Blood samples were taken weekly or every two or four weeks, about 24 h after the last drug intake (trough values), except for the first two samples of patient 1-005 (blood sample taken less than 1 h after drug intake).

### 4.4. Standard and Internal Standard Solution Preparation

A 2,5-DHB stock solution at 100 mg/mL was prepared in MeOH/H_2_O/TFA 0.5:0.5:0.001 (v/v/v). In parallel, stock solutions of osimertinib for the different calibration levels were prepared with serial dilution in MeOH/H_2_O/ TFA 0.5:0.5:0.002 (v/v/v), from 2000 to 10 ng/mL (Table 1) and a stock solution of the IS [^13^C,^2^H_3_]-osimertinib was prepared in MeOH/H_2_O 1:1 (v/v) at 400 ng/mL. A similar workflow was used for the preparation of the QC samples of osimertinib used in the validation batches, with serial dilutions on four concentration levels from 1600 to 10 ng/mL (Table 1).

### 4.5. Sample Preparation

**Figure 6:**
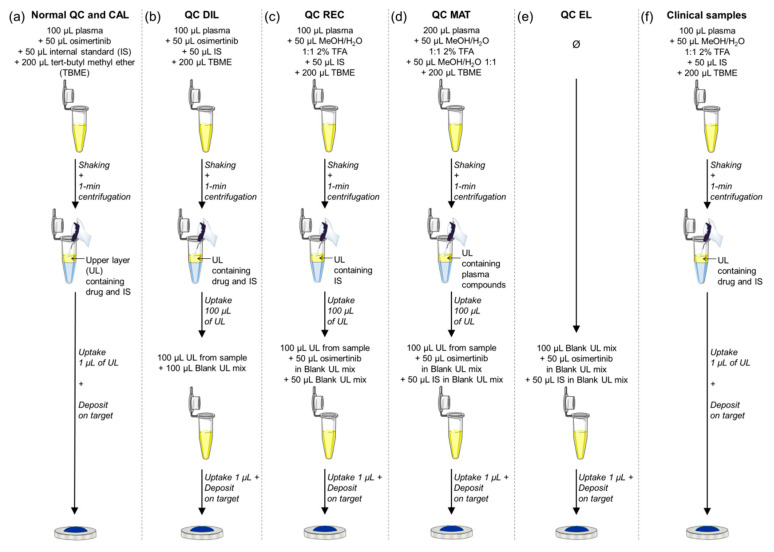
Detailed workflow for the preparation of the quality control (QC), calibration standard (CAL), and clinical samples during validation and analytical batches of the matrix-assisted laser desorption/ionization–ion mobility–tandem mass spectrometry (MALDI–IM–MS/MS) assay for osimertinib quantification. (**a**) Preparation of CAL samples for all batches and normal QC samples for accuracy and precision batches, and for analytical batches. (**b**) Preparation of QC DIL (diluted QC) samples for recovery experiments. (**c**) Preparation of QC REC (QC recovery) samples for recovery experiments (corresponding to the hypothesis of total extraction of osimertinib using the liquid–liquid extraction process). (**d**) Preparation of QC MAT (QC matrix) samples for the matrix effect experiments. (**e**) Preparation of the QC EL (QC eluent) for the matrix effect experiments. (**f**) Preparation of the clinical samples for the analytical batches.

The sample preparation method consisted in a two-step LLE approach that was adapted for the extraction of osimertinib from plasma in a low volume (Figure 6a). For this, 100 µL of plasma was mixed with 50 µL of the reference solution at the desired concentration and 50 µL of the IS solution at its fixed concentration. This resulted in virtual concentrations in plasma from 1000 to 5 ng/mL for CAL samples and from 800 to 5 ng/mL for QC samples (Table 1). In the spiked plasma, 200 µL of TBME was added and the batches of samples vigorously shaken for 10 s. The samples were centrifuged for 1 min at 13,200× *g*. Two layers were clearly distinguished. For the deposition of the samples, two approaches were followed. A first approach consisted in the deposition of 1 µL from the upper layer on the MALDI metal target, followed by the deposition of 1 µL of the 2,5-DHB solution. The second approach consisted in pre-spotting 1 µL of the 2,5-DHB solution on the MALDI metal target. After crystallization, 1 µL of the upper layer from the LLE was deposited on the crystallized spots of 2,5-DHB. After dryness and 2,5-DHB recrystallization, the samples were analyzed.

### 4.6. MS Parameters

The analyses were performed using a Synapt G2-Si instrument (Waters Corp, Milford, USA) consisting of an orthogonal acceleration (oa)-quadrupole (Q)–IMS–time-of-flight (TOF) mass spectrometer equipped with a MALDI source and controlled under MassLynx v4.1 (Waters) as fully described previously [7]. The instrument was either used in sensitivity mode (“V” mode) or in resolution mode (“W” mode). For the analyses in resolution mode, a previously described ion mobility MS/MS method [7] (“Method 4”) was tuned as follows:

Ion source: Laser intensity—300 arbitrary units (a.u.), motion mode—spiral.

Cooling gas: 10 mL/min, trap gas: 2 mL/min, helium cell: 180 mL/min, IMS gas: 90 mL/min. Trap collision energy: 32 V. Transfer collision energy was off for all methods.

Stepwave (SW) parameters. SW1 wave velocity: 300 m/s, wave height: 15 V. SW2 wave velocity: 300 m/s, wave height: 15 V. SW2 DC offset: 25 V, differential aperture 1: 3 V, differential aperture 2: 0 V. Source ion guide: wave velocity: 300 m/s, wave height: 1 V. RF voltages: SW 300 V, ion guide: 350 V.

Quadrupole parameters. MS/MS mode was used with the targeted nominal mass of the compound of interest. The quadrupole low mass (LM) resolution was set to 4.4 a.u.

TriWaveDC parameters. Trap DC entrance: 3 V, bias: 45 V, trap DC: 0 V, exit: 0 V. IMS DC entrance: 20 V, helium cell DC: 50 V, helium exit: −20 V, bias: 3 V, exit: 0 V. Transfer DC: entrance 4 V, exit 15 V.

TriWave parameters. Nitrogen was used as IMS gas. Trap-wave velocity: 311 m/s, wave height: 4 V. IMS variable wave velocity from 1000 to 300 m/s ramping over a full IMS cycle, fixed wave height: 40 V. Transfer-wave velocity: 315 m/s.

The instrument was calibrated before every batch of analyses using a deposit of 20 mg/mL red phosphorus solution in MeOH/H_2_O/TFA 0.5:0.5:0.001 (v/v/v).

### 4.7. Data Processing for MALDI Measurements

Chromatograms and spectra were opened in MassLynx v4.1. Calibration curves were computed in Prism software version 5.01 (GraphPad, LaJolla, CA, USA). In the present study, recently released recommendations for reporting IM–MS measurements [22] were followed. As IMS is used here as a separative method and not for structural analyses, the DT is reported as IMS data. 2D mobility maps (*m*/*z* vs. DT maps) were obtained using Driftscope version 2.9 (Waters).

For IMS data, two methods of data extraction were adopted, MassI and MobA, as previously described [7]. Briefly, MassI consisted in extracting the maximum intensity of the *m*/*z* peaks of interest in the mass spectra combined with the full TIC, whereas MobA consisted in automatically integrating the area of the extracted mobility peaks corresponding to the compounds of interest using MassLynx software [7].

### 4.8. Method Validation

Guidelines of the FDA and EMA for analytical method validation were followed for method validation [9,10]. Four analytical batches were performed over four different days. Each batch consisted of four technical replicates of blind value (BV, not-spiked control plasma), zero-level calibrators (CAL0, control plasma spiked with only [^13^C,^2^H_3_]-osimertinib), and the eight non-zero calibration levels (CAL5 to CAL1000, Table 1), and in eight technical replicates of the four QC levels (LLOQ, LQC, MQC, and HQC, Table 1).

#### 4.8.1. Accuracy and Precision

QC data from the first three validation batches were used to compute within-run and between-run accuracy and precision of the method. Accuracy (% bias) was calculated as the ratio of the difference between the mean measured concentration and the nominal concentration to the nominal concentration. Precision (% CV) was calculated as a percentage of the determined standard deviation of the mean measured concentration. Freeze/thaw stability over three cycles was tested at −20 °C using quadruplicates of LQC, MQC, and HQC levels during the third batch, and freeze/thaw QC data were integrated into the precision and accuracy calculations.

#### 4.8.2. Specificity

To prove the specificity of the assay, control plasma samples from six healthy individuals were evaluated for interfering signals at the analyte and IS fragment drift times.

#### 4.8.3. Matrix Effect and Recovery

To evaluate recovery and matrix effect, a blank upper layer mix was created to mimic the solvent composition of the processed samples. A total of 2 mL of TBME was mixed with 500 µL of the solvent of [^13^C,^2^H_3_]-osimertinib solution (MeOH/H_2_O 1:1 (v/v)) and 500 µL of the solvent of osimertinib solution (MeOH/H_2_O/TFA 0.5:0.5:0.002 (v/v/v)). The solution was mixed and centrifuged at 13,200× *g* for 1 min. Two layers could be distinguished. The upper layer was retrieved (blank upper layer mix) and further used as the solvent to prepare a 400 ng/mL IS solution and QC solutions (LQC, MQC, and HQC levels) for recovery and matrix effect experiments.

For the recovery evaluation, LQC, MQC, and HQC levels were tested, each in four technical replicates. To truly estimate the recovery of the extraction process, two points were crucial for the preparation of the QC samples. First, spiking the QC solution after LLE of plasma dilutes the processed sample. It is then necessary to create diluted QCs (QC DIL) for the recovery experiment. This QC DIL series was prepared in the same way as for accuracy and precision QC (Figure 6b), by spiking 100 µL of plasma with both osimertinib and [^13^C,^2^H_3_]-osimertinib solutions before performing the TBME LLE. Then, 100 µL of the upper layer was retrieved and diluted with 100 µL of the blank upper layer mix introduced above. The second key point to consider is the signal variability between analytical replicates in MALDI–MS measurements. This is due to the ionization process relying on the co-desorption of the analyte with MALDI matrix crystals. For a better data comparison between samples, it is then necessary to first create a matrix/sample deposit as homogenous as possible, as explained in the Results Section 2.1.2, but also to use a signal normalization strategy. To overcome this in the recovery experiments, the osimertinib signal was then normalized to the IS signal as for quantification. For this, the QC REC (QC recovery) series, representing the samples with 100% recovery of the analyte during the extraction, was prepared as follows (Figure 6c). CAL0 samples were prepared and extracted using LLE process. An amount of 100 µL of the upper layer were retrieved, diluted with 50 µL of blank upper layer mix, and spiked with 50 µL of the QC solutions prepared in the blank upper layer mix as detailed above. Signals of osimertinib from QC DIL and QC REC were normalized to the signal of [^13^C,^2^H_3_]-osimertinib in each sample and QC DIL and QC REC normalized signals were compared to evaluate the recovery of the method.

For matrix effect evaluation, two series of QC were prepared (Figure 6d,e): QC MAT (QC matrix) and QC EL (QC eluent), with the three QC levels (LQC, MQC, and HQC) in four technical replicates. The QC MAT series was prepared as follows: BV samples prepared from 200 µL of control plasma were extracted as described above using LLE, and 100 µL of the upper layer was retrieved and spiked with 50 µL of both QC and IS solutions prepared in the blank upper layer mix as detailed upon. Using 200 µL of plasma instead of 100 µL in normal samples enabled us to overcome the dilution effect occurring by spiking the compounds of interest after the extraction. The QC EL series was prepared by spiking 100 µL of the blank upper layer mix with 50 µL of both QC and IS solutions also used for the QC MAT series. The mobility peak area of osimertinib and [^13^C,^2^H_3_]-osimertinib were compared between QC MAT and QC EL to evaluate the matrix effects of each compound, and the matrix effect of osimertinib was normalized by the matrix effect of [^13^C,^2^H_3_]-osimertinib.

#### 4.8.4. Carry-Over Effect

Potential carry-over was evaluated in the fourth batch. For this, 2,5-DHB solution was deposited on a MALDI metal target previously used for one of the first validation batches, on positions where the highest calibration standards (CAL500 and CAL1000) were measured. One µL of the blank upper layer mix introduced in Section 4.8.3 was deposited to dissolve 2,5-DHB crystals and recrystallize it. The positions were then measured to evaluate the level of signal corresponding to osimertinib.

### 4.9. LC–MS/MS-Based Quantification of Osimertinib

For the extraction of osimertinib from plasma, a LLE method was adapted [20]. Briefly, standard solutions of osimertinib were prepared to obtain the identical virtual plasma concentrations in CAL samples as for MALDI–IM–MS/MS (Table 1) with the exception of CAL5, which was replaced by two other points, CAL1 (1 ng/mL) and CAL2 (2 ng/mL). Briefly, 100 µL of plasma was mixed with 25 µL of IS solution and 25 µL of CAL solution and 100 µL borate buffer pH 9 were added. The samples were vortexed for 5 s and 3 mL of TBME was added. The samples were then shaken for 10 min and centrifuged for 10 min at 3000× *g*. A total of 2 mL of the upper layer was recovered and dried with nitrogen for 10 min at 40 °C. The samples were resuspended in 100 µL acetonitrile (ACN)/H_2_O/FA 0.1:0.9:0.0001. The analyses were performed using the ultra-performance LC (UPLC)–MS/MS system consisting of an Acquity UPLC system (Waters) coupled to a triple-stage quadrupole mass spectrometer (TQD, Waters). For LC separation, eluent A was composed of ACN/H_2_O/FA 0.05:0.95:0.0001 and eluent B composed of ACN/FA 1:0.0001 and a 5-min gradient program was performed. Ionization parameters were as follows: capillary voltage 0.5 V, cone voltage 40 V, cone gas flow (N_2_) 50 L/h, desolvation temperature 450 °C, desolvation gas flow (N_2_) 900 L/h, source temperature 150 °C. Argon was used as collision gas for collision induced dissociation (CID). The following MS/MS transitions were measured in positive ionization mode for MRM mode, with 35 V of collision energy: *m*/*z* 500.3 > *m*/*z* 72.1 for osimertinib and *m*/*z* 504.3 > *m*/*z* 72.1 for [^13^C,^2^H_3_]-osimertinib. The calibration curves and the quantifications were performed using TargetLynx (Waters).

### 4.10. Cross-Validation of MALDI–IM–MS/MS

The use of new bioanalytical technologies is encouraged by the FDA with the conditions that the data obtained with these new platforms should be cross-validated with data from other more classical methods, such as LC–MS/MS [9]. It is then recommended to assess the output of both methods with a minimum of 20 incurred samples, and the acceptance criteria of incurred sample reanalysis should then be applied (for at least 67% of incurred samples should the difference between the two compared methods be within ± 20% of the mean). Osimertinib was here quantified in 25 samples originating from three patients with NSCLC, using MALDI–IM–MS/MS and LC–MS/MS. MALDI samples were processed as described in Section 4.5 with 50 µL of MeOH/H_2_O/TFA 0.5:0.5:0.002 (v/v/v) added before TBME extraction instead of osimertinib solution for volume compensation (Figure 6f). Likewise, LC samples were prepared as described in Section 4.9 with 25 µL of ACN/H_2_O 1:1 added before TBME extraction instead of 25 µL of osimertinib solution. The correlation of quantification results was evaluated using a Pearson product–moment correlation test.

## 5. Conclusions

In this methodological study, we developed a MALDI–IM–MS/MS method for the rapid and sensitive absolute quantification of osimertinib in plasma from patients with NSCLC. To the best of our knowledge, the present workflow is the very first MALDI–IM–MS method to be fully validated according to the guidelines from the FDA and the EMA for bioanalytical method validation. We demonstrated that the method displays comparable quantitative performance as with an LC–MS/MS-based method. Besides providing a readily applicable method to monitor exposure to osimertinib in NSCLC patients, these developments open new perspectives for MALDI–MS and especially MALDI–IM–MS for the reliable rapid quantification of small compound inhibitors in biological fluids.

## Figures and Tables

**Table 1:** Concentrations of the Calibration Standard (CAL) and Quality Control (QC) Samples of Osimertinib in Solution and in Spiked Plasma.

Calibration Point	Concentration in Solution (ng/mL)	Virtual Concentration in Plasma (ng/mL)
CAL1000	2000	1000
CAL500	1000	500
CAL200	400	200
CAL100	200	100
CAL50	100	50
CAL20	40	20
CAL10	20	10
CAL5	10	5
CAL0	0	0
Blind value (BV)	0	0
High QC (HQC)	1600	800
Middle QC (MQC)	800	400
Low QC (LQC)	30	15
Lower limit of quantification (LLOQ)	10	5

**Table 2:** Validation Summary of the Osimertinib MALDI-IM-MS/MS Quantification Assay.

Validation Components	Summary					
Analyte	Receptor tyrosine kinase inhibitor, osimertinib					
Internal standard	[^13^C,^2^H_3_]-osimertinib					
Biological matrix	Human plasma					
Sample volume	100 µL					
Analytical method	MALDI–IM–MS/MS, 1 µL spotted on target, 1-min MALDI analysis.					
Sensitivity	LLOQ at 5 ng/mL, eight replicates in three batches.					
	Precision (% CV)			Accuracy (% bias)		
Within-batch	12.6	15.0	9.8	−3.4	−1.6	4.6
Batch-to-batch	11.9			0.7		
Specificity	No detectable interfering peak for osimertinib (0.0% of lowest LLOQ signal) and [^13^C,^2^H_3_]-osimertinib (<0.4% of [^13^C,^2^H_3_]-osimertinib mean signal) observed in blank human plasma samples from six individual sources.					
Carry-over	No significant mean interfering signal for osimertinib (<17.0% of lowest LLOQ signal) and [^13^C,^2^H_3_]-osimertinib (<0.4% of [^13^C,^2^H_3_]-osimertinib mean signal) observed in pure 2,5-DHB samples following CAL500 and CAL1000 standards.					
Calibration range	5–1000 ng/mL					
QC within-batch results	Precision (% CV)			Accuracy (% bias)		
LQC—15 ng/mL	5.3	7.4	9.1	2.7	1.0	−5.9
MQC—400 ng/mL	3.2	3.2	3.5	2.4	−0.7	−0.7
HQC—800 ng/mL	4.5	5.0	1.8	3.5	0.6	2.3
QC batch-to-batch results	Precision (% CV)			Accuracy (% bias)		
LQC—15 ng/mL	8.4			−1.7		
MQC—400 ng/mL	3.5			0.2		
HQC—800 ng/mL	3.8			2.2		
Freeze-and-thaw stability	Demonstrated over three freeze-and-thaw cycles from −20 °C to room temperature on the three QC levels in four replicates.					
Recovery	Average of 53.6% for the recovery of osimertinib for the three QC levels in four replicates using TBME liquid/liquid extraction (13.4% CV).					
Matrix effect	Normalized ^1^ matrix-effect consistency for each QC level in four replicates.					
LQC—15 ng/mL	102.5% (9.9% CV)					
MQC—400 ng/mL	98.1% (7.1% CV)					
HQC—800 ng/mL	99.4% (9.3% CV)					

^1^ Osimertinib matrix effect was normalized by [^13^C,^2^H_3_]-osimertinib matrix effect. 2,5-DHB: 2,5-dihydroxybenzoic acid; CV: coefficient of variation; LLOQ: lower limit of quantification; MALDI–IM–MS/MS: matrix-assisted laser/desorption ionization–ion mobility–tandem mass spectrometry; QC: quality control (HQC: high QC; LQC: low QC; MQC: middle QC); TBME: tert-butyl methyl ether.
